# Scientific Items

**Published:** 1887-06

**Authors:** 


### SCIENTIFIC ITEMS.

**On Sugar in the Blood.**—A series of scientific and profound researches has been completed by Prof. Seegen, of Vienna, which has as its result the establishment of the fact that sugar formed by the liver is derived from albumen and fat.

A portion of the results obtained were published sometime ago, and the general conclusions of the whole work are as follows:

1. That the blood passing from the liver contains an infinitely greater quantity of sugar than that entering the organ.

2. The newly formed sugar in the liver is wholly independent of saccharine food, as well as the carbo-hydrates introduced with the food.

3. Even the liver glycogen is unconcerned in the production of sugar in the liver.

4. Albumen and fat are the materials out of which the liver forms sugar

The fact that sugar is formed from fat is a new one, and is not in accord with the previously entertained chemical and physiological ideas. It appeared to the author, therefore, of much interest to experimentally demonstrate the conversion of fat into sugar. This was accomplished by bringing together fatty bodies and blood with finely divided liver substance. The settlement of this question, that sugar is formed from fat by the liver, seems to point to that organ as the great laboratory in which the food is changed for the purposes of life, for the performance of work and the production of heat. It has a great practical significance, inasmuch as it teaches us the full worth of fat as material for food.—*Medical Review.*

**Does Consciousness Continue after Decapitation?**—On the 31st of January, Mr. Hayem read an interesting communication before the French Academy of Sciences upon the effect of transferring the blood of the horse into the head of animals that have just been decapitated. He finds in the first place, that when the head of a dog is suddenly severed from the body, the eyes for sometime execute motions of anxiety, the jaws forcibly open and close, the eyes afterward become fixed, the nostrils dilate, the labial commissures contract, and the tongue contracts to the back of the mouth A few seconds later, a few respiratory efforts are remarked, and finally the head becomes inert. These phenomena, as a whole, never last more than two minutes.

If, as soon as the decapitation has been effected, the carotids are put in communication with the cubital artery of the horse, the vital manifestations are observed to last half an hour. Finally, if the transfusion is not effected until the head has become inert, the various vital manifestations mentioned above reappear, but definitely cease at the end of a few minutes.

Mr. Hayem concludes that the extinction of will and sensation is very rapid, if not immediate. Conscious life may, nevertheless, be prolonged for a moment by transfusion, when the operation is performed immediately after decapitation; but in the case of the recall to life of an inert head, nothing but automatic motions, without any trace of will, or consciousness even, can be perceived.

Analogous experiments had already been performed by the learned physiologist Brown-Sequard, who came to the conclusion that the will returns to the head of a dog freshly severed from the body. Since then, this opinion has been submitted to no sort of verification. The experiments of Mr. Laborde with human heads that had been severed from the body for over an hour were performed under conditions where success was an impossibility.—*Scientific American.*

**Asbestos Paper.**—Mr. Ladewig has devised a process of manufacturing from asbestos fiber a pulp and a paper that resists the action of fire and water, that absorb no moisture, and the former of which (the pulp) may be used as a stuffing and for the joints of engines.

The process of manufacture consists in mixing about 25 per cent. of asbestos fiber with about from 25 to 35 per cent. of powdered sulphate of alumina. This mixture is moistened with an aqueous solution of chloride of zinc. The mixture is again washed, and then treated with a solution composed of 1 part of resin soap and 8 or 10 parts of water mixed with an equal bulk of sulphate of alumina, which should be as pure as possible. The mixture thus obtained should have a slightly pulpy consistency. Finally, there is added to it 35 per cent. of powdered asbestos and 5 to 8 per cent. of white barytes. This pulp is treated with water in an ordinary paper machine and worked just like paper pulp.

In order to manufacture from it a solid cardboard, proof against fire and water, and capable of serving as a roofing material for light structures, sheets of common cardboard, tarred or otherwise prepared, are covered with the pulp. The application is made in a paper machine, the pulp being allowed to flow over the cardboard. Among other uses, the asbestos paper has been recommended for the manufacture of cigarettes.—*L' Industrie.*

**A Wonderful Advance in the Application of Electricity.**—Dr. C. W. Chancellor, of Baltimore, in *Maryland Med. Journal*, refers to a wonderful apparatus invented by Dr. Roisseau du Rocher, which removes all difficulty in the diagnosis of certain pathological conditions of the stomach, rectum and bladder. It is an instrument armed with an electric light, by which the interior of the organs mentioned may be readily examined by the eye, and the inner walls of the stomach photographed, if need be. The existence of cancer or ulcer in the stomach or rectum can be diagnosed with as much certainty as if it existed on the outer surface of the body, and a stone can be seen as distinctly in the bladder as if it were in the palm of the open hand. It is stated that this instrument can be used by any physician after a little practice, and when it is once seen and the *modus operandi* explained, there can no longer exist in the mind of any intelligent person a doubt as to its value and efficacy, in diagnosing diseases of either the stomach, rectum or bladder.

